# Designing a rheumatology and immunology nursing training model for resource-limited settings: application of the Medical Research Council framework

**DOI:** 10.3389/fmed.2026.1742971

**Published:** 2026-03-23

**Authors:** Lijuan Xia, Fang Feng, Yuan Ma, Bin Yan, Mei Yang, Jijuan Yang, Shuhong Chi, Li Fang, Jie Li, Dongmei Huang, Chenyan Lou, Zhaolan Xia, Jing Mei, Lihong Mai, Yuke Xia, Naoko Hayashi

**Affiliations:** 1 Department of Radiotherapy, General Hospital of Ningxia Medical University, Yinchuan, Ningxia, China; 2 Graduate School of Nursing Science, St. Luke’s International University, Tokyo, Japan; 3 Department of Rheumatology and Immunology, General Hospital of Ningxia Medical University, Yinchuan, Ningxia, China; 4 Nursing School, Ningxia Medical University, Yinchuan, Ningxia, China; 5 Ningxia Fifth People's Hospital, Shizuishan, Ningxia, China; 6 Department of Rehabilitation and Nursing, Ningxia Vocational and Technical College for Minorities, Yinchuan, Ningxia, China; 7 Department of Traditional Chinese Medicine, People's Hospital of Pingluo County, Shizuishan, Ningxia, China; 8 Department of Nursing, Traditional Chinese Medicine Hospital of Yinchuan City, Yinchuan, Ningxia, China; 9 Department of Nursing, Ningxia Hui Autonomous Region Traditional Chinese Medicine Hospital and Research Institute, Yinchuan, Ningxia, China; 10 Department of Oncology, Wuzhong People’s Hospital, Yinchuan, Ningxia, China; 11 College of Clinical Medicine, Ningxia Medical University, Yinchuan, Ningxia, China

**Keywords:** Case-Based Learning, core competences, education models, nursing education, research methodology, rheumatology, immunology

## Abstract

**Background:**

Inadequate training has intensified the shortage of specialized rheumatology and immunology nurses, particularly in resource-limited settings. To address this gap, a theory-driven and evidence-based training model was developed to strengthen nurses’ core competencies and professional capacity.

**Methods:**

The model was developed in two sequential phases following the updated Medical Research Council (MRC) framework for complex interventions. Phase 1 focused on evidence identification through five activities: evidence synthesis, stakeholder engagement, program theory development, contextual analysis, and economic evaluation. Phase 2 involved a two-stage modeling process, including the construction of a hierarchical case library and the design of a tiered Case-Based Learning (CBL) model incorporating progressive levels of complexity (basic, intermediate, and advanced).

**Results:**

Evidence synthesis identified CBL as the most effective educational strategy for enhancing nursing competencies, with progressive implementation showing particular promise. Stakeholder engagement revealed low trainee confidence and dissatisfaction with current unsystematic programs, while trainers emphasized the need for structured CBL, laboratory test interpretation, medication management, and communication skills. The program theory, grounded in the Zone of Proximal Development, supported a staged learning pathway from foundational to advanced levels. Contextual analysis outlined key barriers and six design elements to address them, and the economic evaluation confirmed the feasibility and cost-effectiveness of a localized training model. The final product was a hierarchical case library constructed from authentic clinical records and a three-tiered CBL model ensuring continuity through a spiral progression of knowledge and skills.

**Conclusion:**

This study developed a theory-driven, evidence-based nursing training model using the MRC framework and tailored to local needs in resource-limited hospitals. The model offers a structured and scalable approach to strengthening competencies in rheumatology and immunology nursing and provides a methodological reference for developing complex educational interventions in similar settings. A pilot cluster-randomized controlled trial has been conducted to further evaluate its feasibility and effectiveness.

## 1 Introduction

Rheumatic and immunologic diseases rank as the third leading cause of death globally, following cardiovascular diseases and malignancies (1). This category encompasses over 200 conditions, which are typically complex and varied, affecting multiple organs and systems and characterized by frequent, recurrent episodes (2). These diseases not only affect a substantial patient population but also have prolonged courses, high disability rates, and increased mortality. Additionally, many patients suffer from multiple concurrent rheumatic and immunologic diseases, further complicating management (3). Consequently, these diseases impose significant physical and mental burdens on patients, severely impacting their quality of life and contributing to considerable societal economic losses (4).

For instance, in China alone, more than 200 million individuals are affected, with millions suffering from related conditions like rheumatoid arthritis, systemic lupus erythematosus, spondylarthritis, and gout (5). However, surveys indicate a critical gap in medical staff-patient ratios, leading to inadequate care and resource distribution (6). Specifically, according to the 2024 Ningxia Hui Autonomous Region National Economic and Social Development Statistical Bulletin published by the Ningxia Bureau of Statistics (official regional statistical authority), the permanent resident population of Ningxia was approximately 7.29 million at the end of 2024 (7). Expert assessments further predict a shortage of approximately 350 specialized nurses in rheumatic and immunologic diseases in the autonomous region over the next 5 years, highlighting an urgent need for targeted training programs (8).

Core nursing competencies are fundamental to clinical effectiveness, directly influencing the quality of care and professional development (9). However, there is no standardized, comprehensive training model for nurses in this specialty. Training is particularly challenging due to the complex pathogenesis and abstract immunological concepts associated with these diseases. Many related rare diseases are inadequately covered in existing resources, and the field is advancing rapidly, further complicating the learning process.

To address these challenges, innovative training methods are essential to enhance nurses’ core competencies. One promising approach is Case-Based Learning (CBL), an educational strategy that strengthens problem-solving skills through real-case analysis, reflection, and discussion (10). Since its introduction in 1908, CBL has been widely applied in medical education. Research suggests that CBL effectively shifts learners from passive recipients to active constructors of knowledge by integrating theoretical learning with practical application (11). This approach improves engagement, optimizes resource utilization, enhances educational outcomes, and fosters critical thinking skills.

Building on traditional CBL, Progressive Case-Based Learning (Progressive CBL) uses interconnected and logically sequenced cases to elucidate specific knowledge points (12). This method sustains trainees’ interest in case analysis and problem-solving while promoting active learning and systematic thinking (13). Given the complexity and diversity of rheumatic and immunologic diseases, progressive CBL may provide an effective framework for structured nurse training. Its flexible and nonlinear design allows for tailoring complex interventions (14), which is particularly important for the intricate nature of nurses’ training.

Therefore, to systematically integrate innovative teaching methods into a structured training model, we adopted the Medical Research Council (MRC) framework. This framework is well-suited for managing complex and interrelated training components (15), ensuring both effectiveness and adaptability to the evolving educational needs of nurses specializing in rheumatology and immunology (16). The MRC framework outlines four key phases for complex interventions: development, feasibility/piloting, evaluation, and implementation (17).

In line with this methodological guidance, the present study focused on the development phase of the updated MRC framework. This first phase emphasizes identifying theoretical foundations, synthesizing existing evidence, consulting stakeholders, and modeling potential training strategies prior to implementation (18). Such a systematic approach is particularly important in rheumatology and immunology nursing, where diverse learning needs and complex disease mechanisms require carefully tailored educational designs.

The purpose of this manuscript is not only to present a theory-driven, evidence-based training model for rheumatology and immunology nurses, but more importantly, to illustrate the methodological process of developing a complex educational intervention. By documenting how progressive CBL can be integrated within the updated MRC framework, we aim to provide a practical example of applying this structured approach in nursing education. Highlighting the methodology represents the distinctive contribution of this work, offering insights that may inform the design of other complex educational interventions in specialized healthcare field methods and are suitable for resource-limited settings.

## 2 Methods

### 2.1 Study design and oversight

**Figure 1:**
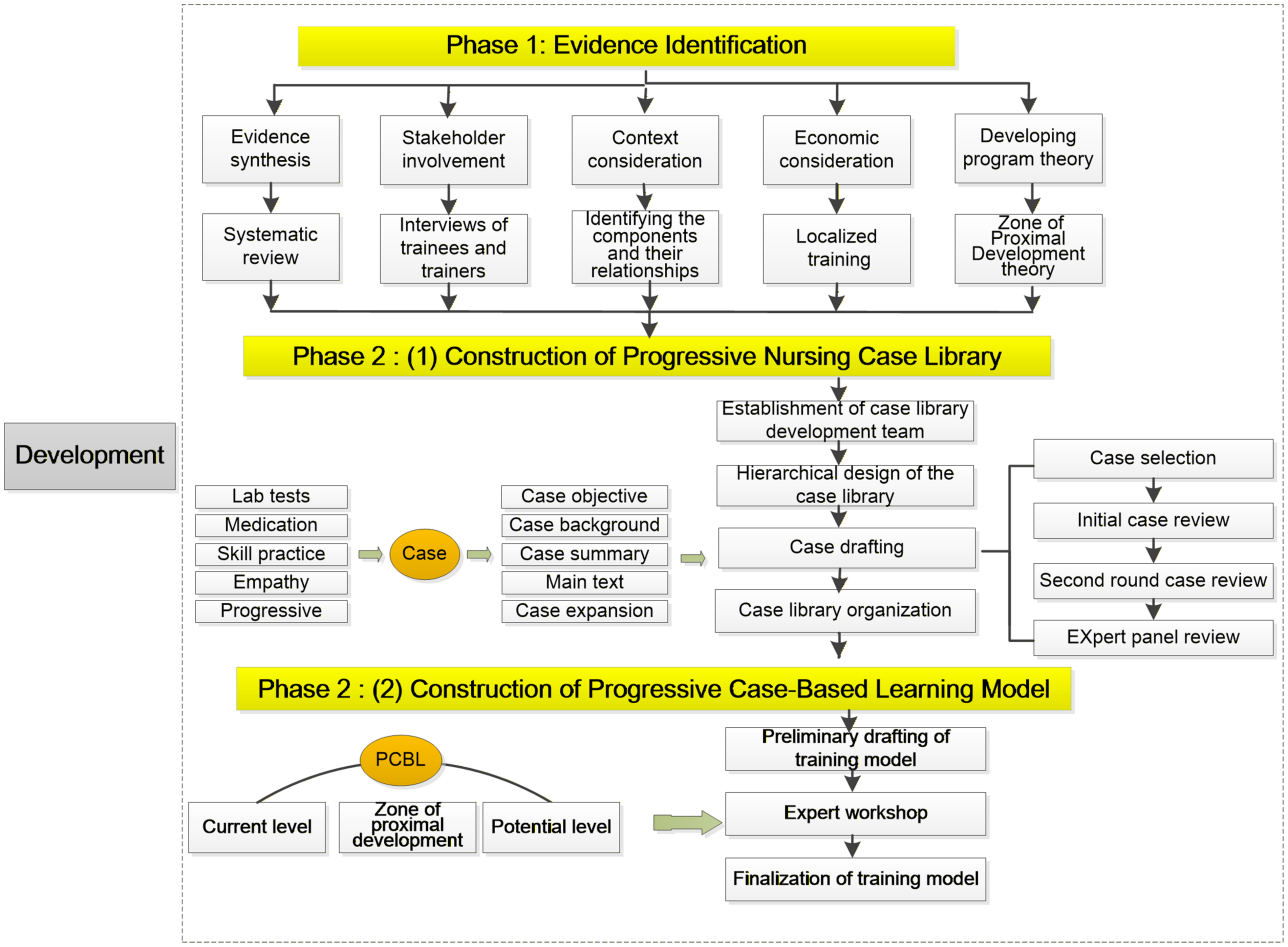
Overview of the development process for the training model following the MRC framework. The process includes two main phases: (1) Evidence identification (evidence synthesis, stakeholder engagement, program theory development, contextual analysis, and economic evaluation) and (2) A two-stage modeling process (construction of a hierarchical case library and development of a tiered CBL model). PCBL, Progressive Case-Based Learning.

The development of the training model was guided by the updated Medical Research Council (MRC) framework for complex interventions and was conducted in two sequential phases. Phase 1 focused on evidence identification, which included five key activities: (a) evidence synthesis, (b) stakeholder engagement, (c) program theory development, (d) contextual analysis, and (e) economic evaluation (19). Phase 2 involved a two-stage modeling process, consisting of (a) the construction of a hierarchical case library with progressively challenging cases and (b) the development of a tiered CBL model—basic, intermediate, and advanced—that incorporated increasingly complex knowledge domains (Figure 1).

The study protocol was approved by the Ethics Committee of the General Hospital of Ningxia Medical University (Approval No. KYLL-2022-1155). All participants provided written informed consent before participation.

### 2.2 Phase 1: evidence identification

#### 2.2.1 Evidence synthesis

A systematic review was conducted to address the research question: *What training programs have been developed to strengthen rheumatology and immunology nursing competencies?* Nine electronic databases were searched—MEDLINE, PubMed, CINAHL, EMBASE, Cochrane Library, Web of Science, China National Knowledge Infrastructure, Chinese Biomedical Database, and Wan Fang. The search strategy combined keywords related to *“training,” “rheumatology,” “immunology,” “specialist nurses,”* and *“practice nurses.”* Searches were limited to studies published in English or Chinese between April 2000 and April 2023. The search strategy was collaboratively developed and validated by a specialist health sciences librarian. As no rheumatology- or immunology-specific nursing training programs were identified, the scope was subsequently expanded to include general nursing training interventions using CBL approaches within the same time and language limits.

#### 2.2.2 Stakeholder involvement

Semi-structured interviews were conducted with trainees and trainers to inform the development of the training model. Purposive sampling continued until thematic saturation was achieved. The trainee group included 15 nurses with ≤5 years of experience from rheumatology and immunology departments across Ningxia Province, selected based on prior survey results to ensure diversity in baseline competencies. The trainer group comprised 11 head nurses and specialist instructors involved in specialty supervision and training. Interview guides were developed through literature review and team discussions, piloted with three participants, and refined with input from two nursing education experts. Questions for trainees explored previous training experiences, perceived usefulness, and improvement needs, whereas trainer questions focused on teaching experiences, perceived trainee outcomes, and program design recommendations.

Interviews were conducted between April and June 2022 in private settings, each lasting approximately 35–40 min. One moderator facilitated each session while an observer recorded field notes. All interviews were audio-recorded and transcribed verbatim, and data were analyzed using Reflexive Thematic Analysis (20). Two researchers independently coded transcripts and iteratively developed subthemes and overarching themes, resolving discrepancies through discussion. Selected excerpts were translated and back-translated to ensure linguistic and conceptual accuracy.

#### 2.2.3 Context consideration

A problem-focused contextual analysis was conducted to identify barriers and enabling factors influencing the implementation of training in resource-limited settings. The analysis integrated evidence from the literature with insights from stakeholder interviews to develop a requirements specification for model design, including delivery format, content scope, assessment strategies, and implementation support mechanisms.

#### 2.2.4 Economic consideration

An economic feasibility assessment was conducted to compare two potential delivery models: a centralized program implemented at a teaching hospital and a decentralized program delivered at county-level hospitals. From a provider perspective, direct costs (facilities, materials, accommodation, travel, trainer fees) and indirect costs (logistics, staff time) were itemized using hospital financial records, standard cost schedules, and administrative interviews. The cost per trainee was calculated for each model, and a comparative scenario analysis was performed to inform the selection of the most feasible and scalable approach for Phase 2.

#### 2.2.5 Developing a program framework

Findings from the systematic literature review and stakeholder interviews were synthesized into a conceptual framework informed by Vygotsky’s Zone of Proximal Development (ZPD) (21). The framework was developed collaboratively and iteratively by the research team (22). ZPD was selected as the theoretical foundation because it highlights the dynamic gap between a learner’s current abilities and potential for growth. Accordingly, training strategies were designed to provide structured scaffolding that supports learners within this zone, addressing existing skills, knowledge gaps, and opportunities for progression. This framework served as the pedagogical foundation that guided the subsequent design, modeling, and validation of the rheumatology and immunology nursing training program.

### 2.3 Phase 2: two-stage modeling process

#### 2.3.1 Construction of the progressive nursing case library

A multidisciplinary development team was established, comprising five rheumatology and immunology nursing specialists, one chief physician, one nursing education expert, and two postgraduate students. The experts (excluding students) each had ≥15 years of professional experience, held at least a bachelor’s degree, and possessed a rank of associate professor or higher.

Standardized criteria were applied to guide case selection and formatting, which included a background, summary, and main text. Quality-assurance procedures ensured progressive difficulty, clinical authenticity, and pedagogical relevance. The final case library comprised authentic inpatient cases collected from the Rheumatology and Immunology Department of a tertiary Grade-A hospital in Ningxia, China (2019–2023), with all patient data fully de-identified.

Each case included five components: (1) objectives (learning objectives, study checklist, critical-thinking questions); (2) background (themes, definitions, etiology, epidemiology); (3) summary (keywords, course of illness, treatments, key nursing strategies); (4) main text (assessment, diagnosis, treatment, nursing interventions); and (5) case expansion (scenario settings, key assessment points, knowledge extension, and suggested resources). Cases were organized into primary, intermediate, and advanced levels to promote stepwise competency development. All cases underwent two rounds of internal review followed by expert panel evaluation. During the review process, experts examined content sequencing, difficulty calibration, and completeness, and provided feedback that informed minor revisions to enhance instructional coherence and consistency.

#### 2.3.2 Construction of progressive Case-Based Learning model

Building on the case library and guided by the ZPD framework, a progressive CBL model was developed to align with trainees’ developmental needs. The model incorporated a spiral sequence of knowledge and problem-solving tasks, enabling each level to build upon the previous one and fostering incremental growth in both clinical and psychosocial competencies.

Model development proceeded through three key steps: (1) Identifying core teaching content and aligning it with case-specific learning objectives; (2) Structuring content by complexity, ensuring that anchor concepts were revisited across interlinked clinical scenarios to reinforce understanding; and (3) Embedding professional values, including empathy, patient dignity, and professional responsibility within each case activity.

To validate and refine the model, four expert workshops (120 min each) were convened with 12 regional and national experts (eligibility: bachelor’s degree or higher, deputy senior title or above, and ≥10 years of experience in nursing education or clinical training). Experts received the draft model 1 week in advance. Each workshop followed a structured critique process to refine case sequencing, placement of teaching points, scenario presentation, assessment design, and level-appropriate complexity.

## 3 Results

### 3.1 Phase 1: evidence identification

#### 3.1.1 Evidence synthesis

The systematic review initially identified 896 records. After removing duplicates and screening titles, abstracts, and full texts, six randomized controlled and quasi-experimental studies employing CBL were included. The methodological quality of the included studies was generally fair, with common limitations related to random sequence generation, allocation concealment, and blinding. Across studies, CBL was consistently associated with improvements in nursing core competencies, critical thinking, and clinical decision-making performance (23).

#### 3.1.2 Stakeholder engagement

A total of 26 participants were interviewed, including 15 trainees and 11 trainers. Reflexive thematic analysis identified three major themes for trainees and three for trainers (Table 1).

**Table 1:** Training perceptions and expectations of trainees and trainers.

Themes	Sub-themes	Selected quotes
Results of interviews with trainees		
Complexity of the disease	Different clinical manifestations	N4: “These diseases are very complex. For example, among patients with systemic lupus erythematosus, each of the 20 patients can exhibit 20 different clinical manifestations. I was really shocked when I first came to this ward.”
	Many comorbidities	N2: “Before encountering these types of diseases, I did not realize that a patient with rheumatoid arthritis could have so many comorbidities, such as interstitial pneumonia, hypertension, coronary heart disease, diabetes, and depression. All of these factors contribute to the complexity of the case.”
	Complex medication regimen	N5: “Due to the complexity of the disease, medication management is intricate and involves numerous considerations. A single patient may be prescribed more than a dozen different medications, including antibiotics, anti-inflammatory and analgesic drugs, immunosuppression, hydrocortisone, biologics, antihypertensive drugs, hypoglycemic agents, lipid-lowering drugs, calcium supplements, Calcium absorption-promoting drugs and drugs to protect the gastric mucosa.”
	Complicated lab tests	N4: “Lab tests in this field are quite complex. For instance, drawing blood in the morning often requires 20–30 vacuum blood tubes per patient, and some tests are unique. Honestly, I’m not sure of the purpose or indications for some of these tests.”
Insufficient foundational knowledge	Lack of targeted on-the-job training	N11: “Our hospital is relatively small, and the rheumatology ward is combined with the nephrology ward. As a result, I feel there has been a lack of foundational training in rheumatology.”
	Unsystematic training	N6: “Without systematic training, my current understanding of rheumatology feels disorganized. The training I received was not structured step-by-step; instead, some of the more complex concepts were introduced first, making them difficult to grasp and resulting in poor outcomes.”
	Unmotivated training	N7: “I find the training to be somewhat unengaging. The material is complex and presented in a way that fails to capture my interest, which decreases my motivation to participate.”
Key training suggestions	Incorporate educational engagement	N5: “It would be advantageous to design training that is both educational and engaging. The previous sessions were tedious and overly complex, leading to frustration. By incorporating interactive elements, real-world examples, and practical applications, the training could become more effective and enjoyable.”
	Step-by-step training	N13: “The previous training was overly complex. We need to start with the basics and progressively build up to more advanced concepts. I feel the current training in the department has not achieved its goal and followed this approach. “
	Incorporate clinical skill practice	N10: “In previous training sessions, instructors frequently delivered specialized knowledge without assessing our understanding. Consequently, we felt uncertain about how to apply this knowledge when needed. Future training should include practical sessions to ensure that participants truly grasp and can apply the knowledge.”
Results of interviews with trainers		
Limited capability	Lack of trainer training	T7: “Rheumatology and immunology are emerging fields, and we have not had systematic training ourselves. This leaves us uncertain about how to provide structured and effective training.”
	Delayed knowledge updates	T2: “The field is evolving rapidly, with new concepts, knowledge, and drugs continuously emerging. We have not had systematic training on these developments ourselves.”
Absence of training plan and materials	Absence of systematic training plan	T1: “Our department does not handle many rheumatology and immunology cases, and we lack a systematic training plan. We only provide training related to specific diseases as we encounter these patients.”
	Absence of practical training materials	T3: “Last year, I found that there were very few relevant reference books or materials available when I tried to create new training plans”
Essential training suggestions	Case-based teaching method	T5: “I believe the case-based teaching method is quite effective. It presents relevant knowledge within the context of cases, which can engage nurses and help them learn and apply the necessary knowledge in practice.”
	Stratified training	T1: “Courses should be designed to progress from basic to advanced, and from simple to complex, in a step-by-step manner. Otherwise, the training may not achieve the desired results.”
	Incorporating medication knowledge	T5: “There are many drugs used in the rheumatology and immunology department. Although we have covered the pharmacology knowledge in training before, the results were not very effective. In case-based teaching, we should focus on explaining each drug individually and emphasize 1–2 drugs after each case. This approach helps create a long-lasting impression and makes it easier for trainees to remember.”
	Including lab test knowledge	T9: “There are numerous lab tests for patients with rheumatological and immunological conditions, but some young nurses are not familiar with their significance and methodologies. Integrating this information into case-based training would be highly beneficial. While excessive detail is not necessary, a solid understanding of the purpose, methods, and precautions of these tests is essential for nursing practice.”
	Incorporating chronic disease management	T8: “Chronic diseases are characterized by recurrent episodes, making it essential to train nurses in managing these conditions and preventing complications. Comprehensive training should include understanding the disease’s progression, recognizing early signs of exacerbations, and implementing effective intervention strategies. Nurses should also be equipped with skills to educate patients on lifestyle modifications, medication adherence, and self-monitoring techniques.”
	Develop content focused on effective communication	T3: “Patients with chronic diseases may experience psychological issues such as suspicion, anxiety, and depression. Training should include strategies for effectively communicating with these patients.”
	Enhancing empathy skills	T10: “Some nurses are relatively young and may find it challenging to empathize with patients and understand their psychological status. To address this, future training should prioritize patient care and respect, focusing on cultivating professional skills and empathy. This includes enhancing communication, active listening, and emotional intelligence, enabling nurses to connect more effectively with patients and deliver comprehensive psychological support.”
	Arrange training time reasonably	T2: “Choosing the right timing and duration for training is crucial. Given the busy nature of clinical work, the design should be practical. A blended approach, combining online and offline teaching, is preferable as it accommodates busy schedules and minimizes time constraints.”

Trainees emphasized: (1) Complexity of diseases: multiple comorbidities, diverse manifestations, complex medication regimens, and extensive laboratory testing; (2) Insufficient foundational knowledge: lack of targeted, systematic, and engaging on-the-job training; and (3) Preference for stepwise, interactive, and practical programs that combine theory with hands-on application.

Trainers highlighted: (1) Limited instructional capacity—inadequate specialty preparation and outdated knowledge; (2) Absence of training plans and materials—lack of systematic curricula and high-quality resources; and (3) Essential improvement recommendations—adoption of stratified CBL, incorporation of pharmacology and lab-test content, chronic-disease management, communication, empathy, and flexible scheduling. These findings confirmed the educational gaps and the urgent need for a structured, progressive, and context-appropriate training model.

#### 3.1.3 Context consideration

The contextual analysis identified four major categories of barriers affecting the delivery of effective rheumatology and immunology nursing training in resource-limited settings: (1) Complex Disease Characteristics: Rheumatologic and immunologic conditions are inherently complex in their pathogenesis, clinical manifestations, and treatment protocols. Abstract immunological concepts are often difficult for nurses to grasp, posing challenges for conventional educational methods (24). (2) Gaps in Educational Resources: Rare but critical conditions such as IgG4-related disease, panniculitis, and vasculitis are frequently absent from standard nursing textbooks and training materials, leaving frontline nurses underprepared for real-world practice (25). (3) Complicated Disease Management: Severe conditions such as hemophagocytic syndrome and catastrophic antiphospholipid syndrome are diagnostically and therapeutically demanding, complicating the design of standard training curricula (26, 27). (4) Rapid Field Advancements: Rheumatology and immunology evolve rapidly, with frequent updates to diagnostic criteria, treatment regimens, and pharmacological options, underscoring the need for lifelong learning and adaptability (28).

To address these challenges, the analysis identified six essential elements for an effective training model:

(1) CBL: CBL enhances clinical reasoning and learner engagement through interactive, scenario-driven instruction. Practical case applications should encompass symptom recognition, diagnostic interpretation, treatment planning, medication management, chronic disease care, and lifestyle interventions. Developing a nursing-specific case repository is essential for contextual relevance (29, 30).
(2) Stratified and Progressive Learning: The training should be organized in a step-by-step manner, aligned with ZPD construct, allowing participants to progressively integrate existing knowledge with new information. A tiered approach ensures that each level builds upon the previous one, maintaining engagement and preventing cognitive and knowledge overload.
(3) Deployment of Qualified Trainers: Local capacity gaps in training delivery necessitate the involvement of well-prepared instructors. A model in which trained team members are dispatched to partner hospitals can optimize resources while improving consistency. A blended learning format, combining online modules with in-person sessions enhances flexibility and reach.
(4) Integrated Knowledge Delivery: The curriculum should present laboratory diagnostics, pharmacology, and chronic disease care not as isolated subjects, but as integrated elements within clinical cases. This reinforces theoretical understanding through practical application and contextual relevance.
(5) Clinical Skills and Decision-Making Practice: Hands-on sessions are vital for developing procedural proficiency and clinical judgment. By simulating real-world scenarios, trainees can gain confidence, refine decision-making, and bridge the gap between theory and practice.
(6) Empathy and Psychological Care Training: Enhancing empathy among young nurses should be a core component of the training. The training should explicitly include components that cultivate empathy and promote holistic, patient-centered care. Addressing the psychosocial aspects of illness not only enhances patient outcomes and communication but also reduces burnout and improves job satisfaction among nurses.

#### 3.1.4 Economic consideration

Economic evaluation demonstrated substantial cost differences between centralized and decentralized delivery models. Training one participant in a teaching hospital was estimated to cost ≈US $1,400, while county-level implementation reduced costs to ≈US $275 per trainee. Considering affordability and scalability, the decentralized model was selected for subsequent implementation.

#### 3.1.5 Developing a program framework

The final conceptual framework was structured according to the principles of ZPD-based learning (31–33). It conceptualized the learning process as a dynamic interaction between instruction and development, in which trainees advance from their current to potential levels of competence through guided support. The framework identified four key principles: (1) learning gaps are shaped by both prior experience and the degree of instructional support; (2) targeted interventions, such as stepwise guidance, feedback, and reflective practice facilitate progression within the ZPD from assisted to autonomous performance; (3) scaffolding strategies should be temporary, adaptive, and gradually withdrawn as competence improves; and (4) effective instructional design requires explicit assessment of each trainee’s baseline to align task complexity with developmental readiness.

By applying these principles, the framework emphasized progressive, learner-centered, and feedback-oriented training, enabling nurses to bridge the gap between current and potential performance. This theoretical structure provided the pedagogical foundation for designing the hierarchical CBL model and guided the sequencing of content and complexity in the final training model.

### 3.2 Phase 2: model development

#### 3.2.1 Construction of the progressive nursing case library

A total of 54 authentic inpatient cases were compiled into a three-tiered hierarchical library—primary, intermediate, and advanced—encompassing a comprehensive range of rheumatologic and immunologic conditions (Table 2). Each case underwent two rounds of internal review followed by expert-panel appraisal to ensure accuracy, pedagogical coherence, and progressive complexity.

**Table 2:** Constructed the progressive nursing case library of rheumatology and immunology.

Primary case library	Intermediate case library	Advanced case library
Rheumatoid arthritis	RA with interstitial pneumonia	RA with depression
Ankylosing spondylitis	Inflammatory bowel disease-associated arthritis	Ankylosing spondylitis with uveitis
Sjogren’s syndrome	SS with interstitial pneumonia	SS with multi-system involvement
Gout	Gout with renal failure	
Scleroderma	Systemic sclerosis	SSc with pulmonary arterial hypertension
Adult-onset Still’s disease		AOSD with macrophage activation syndrome
SLE with hematological system damage SLE with renal impairment SLE with interstitial pneumonia	SLE with heart failure SLE with renal failure SLE with polyserositis SLE with pulmonary fungal infection SLE with pregnancy	SLE with antiphospholipid syndrome SLE with pulmonary hypertension SLE with neurological damage SLE with multisystem damage Overlap syndrome (SLE + SSc + SS) SLE with neuromyelitis optica SLE with hemophagocytic syndrome
Behçet’s disease	Intestinal Behçet’s disease	BD with vascular involvement BD with neurological involvement
Idiopathic inflammatory myopathies	IIMs with interstitial pneumonia IIMs with severe infection IIMs with arrhythmias	IIMs with malignancy IIMs with pulmonary arterial hypertension MDA5-positive dermatomyositis
Large vessel vasculitis		Large vessel vasculitis with aneurysm
Polymyalgia rheumatica		IgG4-related disease
Vasculitis	Wegener’s granulomatosis Microscopic polyangiitis Eosinophilic granulomatosis with polyangiitis	Nodular polyarteritis
Erythema nodosum	Undifferentiated connective tissue disease	
Fibromyalgia syndrome	Mixed connective tissue disease	

RA, rheumatoid arthritis; SS, Sjögren’s syndrome; SSc, systemic sclerosis; AOSD, adult-onset Still’s disease; SLE, systemic lupus erythematosus; BD, Behçet’s disease; IIMs, idiopathic inflammatory myopathies.

During the expert review, several refinements were introduced, including the addition of pre-admission histories and pregnancy outcomes to enhance case realism, as well as reordering of case sequences to optimize the flow of knowledge and difficulty. The finalized case library provided a structured and developmentally aligned foundation for the subsequent training model, ensuring both clinical relevance and educational progression.

#### 3.2.2 Construction of progressive Case-Based Learning model

Building upon the progressive case library and guided by the ZPD framework, a three-level progressive CBL model was developed to match trainees’ developmental stages and clinical learning needs. The model adopted a spiral learning structure, allowing each level to build on the previous one and promote cumulative growth in clinical reasoning, procedural competence, and psychosocial understanding.

Model refinement was achieved through four expert workshops (120 min each) involving 12 regional and national experts. The workshops followed a structured peer-review approach to evaluate the model’s content alignment, pedagogical coherence, and developmental appropriateness. Experts recommended several modifications: (a) reordering six key teaching points to optimize knowledge flow; (b) aligning case complexity with learner proficiency; (c) adding follow-up outcomes to four chronic cases to enhance continuity of care; (d) revising formative assessment rubrics to capture learner progression; and (e) embedding empathy and patient-centered communication objectives in each scenario.

The finalized model was validated by all experts as feasible, coherent, and educationally sound, forming the core instructional framework for specialty training.

#### 3.2.3 Implementation process

Implementation of the progressive CBL model followed a five-step sequence designed to integrate theoretical learning with clinical application:(1) Case distribution: Thematic materials—learning objectives, study checklists, supplemental resources, and critical-thinking prompts—were distributed via the hospital learning portal or WeChat mini program 1 week in advance. Trainees reviewed materials before group discussion. (2) Pre-training assessment: A brief formative quiz, accessed via QR code, assessed baseline understanding of case content and objectives 1 day prior to the session. (3) Case training: During in-class sessions, trainers facilitated structured discussions around assessment findings, nursing challenges, interventions, and complications. Learner presentations and instructor feedback reinforced key learning points. (4) Clinical practice: Designated instructors mentored trainees during ward rotations, guiding real-world application of theoretical knowledge and addressing learning gaps. (5) Post-training assessment: After each case cycle, trainees completed evaluations measuring knowledge retention, practical application, and training effectiveness.

A representative example focused on systemic lupus erythematosus with hematological involvement is shown in Tables 3, 4, illustrating the model’s integration of pre-class preparation, interactive workshops, and post-class consolidation within a unified learning continuum.

**Table 3:** Key design of a case involving systemic lupus erythematosus (SLE) with hematological involvement.

Key element	Key points and main content	Key skill development	Training design
Introduction	Case overview and objectives	Inspire clinical curiosity	Case introduction
Clinical manifestations	Symptoms and signs	Foundational knowledge	Video and image demonstration
Case treatment process	Medication therapy + adverse reaction management	Logical reasoning	Interactive discussion
Case treatment outcome	Therapeutic response and related factors	Clinical reasoning	Interactive discussion
Discussion: key observations	Manifestations, medication efficacy, and adverse events	Analytical thinking	Interactive teaching
Discussion: nursing interventions	Comprehensive and prioritized care	Problem-solving	Guided discussion
Discussion: potential complications	Multisystem and multi-organ involvement	Clinical reasoning	Heuristic questioning
Discussion: Chronic disease management	Medication adherence and adverse reaction management	Problem-solving	Heuristic teaching
Case summary	Immune-mediated chronic Inflammation	Summary	Summary and deduction
Simulation: nursing assessment	Basic and specialized assessments (SLEDAI-2000, nutrition)	Knowledge synthesis	Practical teaching
Simulation: health education	Counseling on diet, activity, and psychological care	Clinical practice	Practical teaching
Reinforcement	Summary of key nursing strategies	Memory consolidation	Reinforcement session
Knowledge expansion	Classification of Coombs’ test and the clinical significance of positive results	Knowledge extension	Heuristic teaching

**Table 4:** Instructional design and assessment framework for the SLE case.

1. Training objectives		
1.1 Knowledge objectives Understand the clinical manifestations, diagnostic investigations, and treatment principles of SLE; be familiar with its epidemiology and prognosis. Understand the immunological markers associated with SLE (anti-dsDNA, C3/C4, ANA). Master the mechanism of action and precautions of the biologic agent Telitacicept. Identify common nursing care needs in SLE, especially skin and mucous membrane management. Understand the structure, scoring method, and clinical significance of the SLEDAI assessment tool. Comprehend the therapeutic role, side effects, and nursing interventions related to corticosteroid treatment. Gain basic knowledge of pregnancy planning, fertility risks, and nursing roles in reproductive counseling for SLE patients. 1.2 Technical objectives Learn the correct technique for subcutaneous injection of Telitacicept. Understand pre- and post-care for renal biopsy procedures. Develop basic skills in completing and interpreting SLEDAI scores from clinical data. 1.3 Competency objectives Identify and respond to common nursing problems in active SLE. Provide evidence-based health education. Recognize and manage complications such as hyperkalemia. Collaborate effectively with healthcare team members. Identify issues related to medication adherence, psychological needs, and fertility concerns in SLE patients. 1.4 Professionalism objectives Practice ethical standards and demonstrate humanistic nursing values.		
Phase 1: Pre-class online learning		
Platform: Internal hospital learning portal/WeChat mini program Learning modules: Overview of SLE: clinical features and diagnostic principles Immunological markers in SLE: anti-dsDNA, ANA, C3/C4 Introduction to SLEDAI: structure, scoring, clinical relevance Telitacicept: mechanism and injection techniques (video) Corticosteroid therapy in SLE: indications and side effect management SLE and fertility: timing, medication safety, counseling considerations Assignments: Pre-class learning checklist (PDF/Word) Online quiz (10 items): multiple choice/true-false, auto-graded Clinical reflection task: learners submit questions based on case preview		
Phase 2: In-person case-based workshop (approx. 2 h)		
Location: Seminar room/skills lab Teaching Methods: Case presentation + group discussion + clinical reasoning + instructor feedback Agenda overview: Case Presentation (5 min): Overview of an SLE patient with lupus nephritis, highlighting lab findings, scoring, and treatment Clinical Knowledge Review (20 min): Interactive Q&A on symptoms, diagnostics, and treatment principles SLEDAI Hands-On Scoring (15 min): Small groups calculate SLEDAI scores using real case data Nursing Care Discussion (20 min): Identification of nursing problems, mucocutaneous care, renal biopsy pre/post care Pharmacological Management (10 min): Video review of Telitacicept injection + discussion of corticosteroid side effect management Reproductive Health Focus (10 min): Case-based discussion on pregnancy planning and safe medication use in SLE Complication Management (5 min): Recognizing and handling hyperkalemia in SLE patients Summary & Feedback (5 min): Instructor recap + learner feedback through mini-interview		
Phase 3: Post-class online consolidation		
Post-training knowledge quiz (15 items, includes clinical scenarios) Online satisfaction and feedback survey Optional Resource Pack: SLEDAI-2 K scoring sheet, SLE pregnancy management flowchart, drug interaction checklist		
Clinical practice		
On-site mentoring by designated instructors Real-time feedback and bridging of theory-practice gaps		
Assessment strategy		
Component	Weight	Description
Pre-Class Quiz	20%	Assesses initial knowledge base
In-Person Engagement	30%	Participation in discussions and clinical reasoning
Post-Class Quiz	30%	Application of knowledge to clinical scenarios
Clinical performance	20%	Knowledge application and decision making

SLE, systemic lupus erythematosus; anti-dsDNA, anti–double-stranded DNA; C3/C4, complement components 3 and 4; ANA, antinuclear antibody; SLEDAI, Systemic Lupus Erythematosus Disease Activity Index; SLEDAI-2K, Systemic Lupus Erythematosus Disease Activity Index 2000.

## 4 Discussion

This study presents the design and methodological foundation of a progressive case-based training model for rheumatology and immunology nursing, developed in response to the growing need for specialized education in this complex field. Grounded in the updated MRC framework and informed by Vygotsky’s Zone of Proximal Development, the model provides a structured approach to scaffolding as temporary structured support, clinical knowledge, critical thinking, and humanistic care competencies in a stepwise, developmentally aligned manner.

### 4.1 Framework-driven model development

The updated MRC framework was instrumental in guiding the systematic design of the model. By emphasizing theoretical grounding, contextual understanding, stakeholder engagement, and iterative development, the framework enabled the construction of a multifaceted training program tailored to the realities of rheumatology and immunology nursing in resource-limited settings. In line with Skivington et al. and Bleijenberg et al. (14, 34), this approach enhanced the methodological rigor and ensured the training model addressed not only content delivery, but also learning needs, feasibility, and long-term applicability.

The model integrates CBL as a core instructional method, which was identified during the evidence synthesis as particularly effective for promoting active learning, clinical reasoning, and application of knowledge in real-world contexts. CBL enables learners to engage with authentic clinical scenarios, fostering reflection, adaptability, and decision-making skills essential in managing autoimmune and systemic inflammatory diseases (35). These findings are consistent with educational research by Sultana et al. (35) and Tsekhmister (36), supporting the shift from passive to participatory learning modalities in nursing education.

### 4.2 Developmental scaffolding through the ZPD construct

The ZPD construct offered a complementary lens for structuring the model’s tiered case library and progressive learning process. By aligning learning tasks with nurses’ developmental stages and providing appropriate scaffolding, the model facilitates movement from foundational competence toward independent clinical judgment. This developmental alignment supports deeper engagement without cognitive overload, helping learners build confidence and capacity incrementally.

Existing research on ZPD in nursing education, though limited, echoes this model’s rationale. Studies by Coffman et al., Sanders and Welk, and Kantar et al. (21, 37, 38) emphasize the value of instructor-supported growth and structured challenge in facilitating clinical skill acquisition. Drawing from broader educational theory (39, 40), the application of ZPD in this context illustrates its utility in designing effective, learner-centered training models that are responsive to complexity and evolving professional demands.

### 4.3 Contextual integration and model coherence

Incorporating contextual analysis into model development was key to relevance to rheumatology and immunology nursing practice in resource-limited settings. Six foundational elements were embedded into the design: Case-Based Learning, a stratified and progressive learning structure, qualified instructors, integrated knowledge delivery, clinical skills application, and emphasis on empathy and psychosocial care. These components were not treated as isolated features but were woven into each phase of the program, reinforcing both technical and humanistic dimensions of nursing practice.

A pilot cluster-randomized controlled trial was conducted to assess the model’s effectiveness and acceptability (41), with the results reported in a separate paper. This manuscript is dedicated exclusively to detailing the development methodology. By documenting the structured, theory-informed, and context-sensitive design process, this work contributes a replicable blueprint for others aiming to create similar training models, whether in rheumatology, immunology, or other clinical specialties.

Such methodological transparency is especially critical given the persistent gap in chronic disease management in rural areas of China, where rheumatic and immunological conditions are often underdiagnosed and undertreated (42), despite their high burden of disability and mortality. By integrating contextual needs, learner engagement, and structured competency development, this study exemplifies a shift toward more holistic and contextually grounded approaches in healthcare education.

### 4.4 Sustainability considerations

Sustainability is a key consideration when developing complex educational interventions, particularly in resource-limited settings. Consistent with the principles of the updated MRC framework for complex interventions, considerations of implementation, sustainability, and adaptability were incorporated during the design phase of the training model. The MRC framework emphasizes the importance of developing interventions that are not only theoretically grounded but also feasible and adaptable within real-world contexts (43).

In the present study, several design elements were intentionally integrated to support potential long-term implementation, including the use of locally available clinical cases, reliance on trained local instructors, and alignment with existing hospital-based continuing education structures. These features aim to facilitate integration of the training program into routine educational activities while minimizing reliance on external resources.

Nevertheless, the sustainability of complex educational interventions is often shaped by contextual factors during real-world implementation. Factors such as institutional support, organizational priorities, and evolving clinical and educational needs may influence how the model functions over time. Therefore, the training model should be viewed as a dynamic framework that may require ongoing refinement and iterative adaptation during practical application. Future implementation research will be important for evaluating how the model can be maintained, adapted, and embedded within routine nursing education systems to ensure its long-term effectiveness.

## 5 Implications for practice and education

Although this study does not include the outcome data, the design methodology offers three key implications for nursing education and workforce development: (1) Methodological Framework: The integration of the MRC framework and ZPD construct demonstrates a replicable process for designing structured, learner-centered nursing curricula. (2) Training Design in Complex Fields: The use of progressive CBL and developmental scaffolding provides a model for building clinical competence in specialties that require nuanced decision-making and patient-centered care. (3) Feasibility in Resource-Limited Settings: The localized, context-sensitive development process supports adaptability and scale-up in under-resourced training environments. This approach also contributes to the growing literature advocating educational models that are not only evidence-based but also adaptable and responsive to real-world constraints.

## 6 Limitations

This paper focuses on the development methodology and does not present outcomes from the feasibility study. As such, the effectiveness of the model in improving learning outcomes, clinical performance, or patient care is not included. Additionally, the design process was guided by expert consultation and literature synthesis but did not include patient or interdisciplinary perspectives, which may limit the comprehensiveness of the training scenarios. Future studies should incorporate broader stakeholder input and report on both short- and long-term outcomes following model implementation.

## 7 Conclusion

This study presents the development of a progressive, case-based training model for rheumatology and immunology nursing, grounded in theory and evidence and guided by the updated MRC framework and the ZPD construct. The model aims to improve nurses’ competencies through layered Case-Based Learning, structured clinical practice, and situation-specific instructional strategies. This work establishes a foundation for future evaluation and implementation, offering a replicable approach for a competency-based training model in specialized nursing fields. Subsequently, a pilot cluster-randomized controlled trial (RCT) was conducted to evaluate the feasibility and effectiveness of the training.

## Data Availability

The original contributions presented in the study are included in the article/supplementary material, further inquiries can be directed to the corresponding authors.
